# Configurable analog-digital conversion using the neural engineering framework

**DOI:** 10.3389/fnins.2014.00201

**Published:** 2014-07-22

**Authors:** Christian G. Mayr, Johannes Partzsch, Marko Noack, Rene Schüffny

**Affiliations:** ^1^Neuromorphic Cognitive Systems Group, Institute of Neuroinformatics, University of Zurich and ETH ZurichZurich, Switzerland; ^2^Electrical Engineering and Information Science, Chair of Highly Parallel VLSI Systems and Neuromorphic Circuits, Technische Universität DresdenDresden, Germany

**Keywords:** neural network analog digital converter, neural engineering framework, ADC with signal processing, multiple input ADC

## Abstract

Efficient Analog-Digital Converters (ADC) are one of the mainstays of mixed-signal integrated circuit design. Besides the conventional ADCs used in mainstream ICs, there have been various attempts in the past to utilize neuromorphic networks to accomplish an efficient crossing between analog and digital domains, i.e., to build neurally inspired ADCs. Generally, these have suffered from the same problems as conventional ADCs, that is they require high-precision, handcrafted analog circuits and are thus not technology portable. In this paper, we present an ADC based on the Neural Engineering Framework (NEF). It carries out a large fraction of the overall ADC process in the digital domain, i.e., it is easily portable across technologies. The analog-digital conversion takes full advantage of the high degree of parallelism inherent in neuromorphic networks, making for a very scalable ADC. In addition, it has a number of features not commonly found in conventional ADCs, such as a runtime reconfigurability of the ADC sampling rate, resolution and transfer characteristic.

## 1. Introduction

Circuits for analog-digital-conversion (ADC) are at the heart of every integrated circuit (IC) that deals with sensory or other analog input signals. Their performance and characteristics have a large repercussion on the signal processing carried out in the later (usual digital) stages of the IC, as distortions of the signal introduced in the ADC cannot usually be recovered. In general, ADCs because of their analog nature are handcrafted to achieve optimum characteristics for a given application. They usually require a wide range of custom analog circuit components, such as amplifiers, voltage/charge/current converters, integrators, addition/subtraction circuits, threshold switches, etc (van de Plassche, 2003).

However, this handcrafted, analog nature of ADCs is not in keeping with todays mostly digital Systems-on-Chip (SoC). SoCs due to their digital nature can be rapidly prototyped and transferred across technology nodes, something not possible with a handcrafted analog circuit. In addition, state-of-the-art deep-submicron technology nodes have become increasingly worse in their analog performance.

ADCs have started to partially follow this trend, offering architectures such as Delta-Sigma-Modulators (DSM) that only need low-performance analog components and move a large part of their functionality into the digital domain (Marijan and Ignjatovic, 2010; Mayr et al., 2010b). However, to really comply with the demands placed on modern ADCs, inspiration may be taken from a completely different domain, that of neural information processing and neuromorphic design. Neural networks rely for their overall function on multiple replication of a single, simple base element, the neuron. Thus, scaling and technology transfer of a neuromorphic ADC would be simplified. A neural network represents data across a population, thus inherently smoothing out variations and noise and making the signal representation more robust. Neurons take analog data as input, transferring it immediately into a pseudo-digital, timing based pulse representation. Thus, all subsequent processing would be digital directly after this first stage. Neural networks can replicate non-linear transfer functions of one or several input variables (Lovelace et al., 2010). Thus, sensor fusion and analog preprocessing could be achieved, which in conventional ADCs requires separate analog blocks (Chen et al., 2013).

This paper proposes using the Neural Engineering Framework (NEF) (Eliasmith and Anderson, 2004) as a method to build an ADC that incorporates most of the above advantages of neural networks. In the NEF, a signal is encoded across a neuron population by a set of encoder weights and the transfer functions of the neurons. A set of decoder weights can be computed that extracts the signal itself or a transformation of it from the postsynaptic current (PSC) traces of the neurons. By building the encoder step and the neurons in analog circuitry while having the decoding and signal reconstruction done in the digital domain, a straightforward conversion from analog to digital can be established.

Specifically, we show in this paper the usage of NEF as a linear, single input ADC comparable to conventional ADCs. The theoretical and simulative analysis is supported by an example design in a 180 nm CMOS technology, proving feasibility of the approach. The remainder of the paper is structured as follows: section 2.1 introduces the NEF framework. In section 2.2, its general application to analog-digital-conversion is given. Section 2.4 details the analog and digital circuit design. Results are given in section 3.1 for an ADC based on idealized neurons in a neural network simulator. Results for the actual hardware implementation of neurons, encoder and decoder network are given in section 3.2. Section 4 discusses the significance of the results.

## 2. Materials and methods

### 2.1. Representation of analog variables in the neural engineering framework (NEF)

At its most basic, the NEF describes the transmission of an analog value or a set of values across a neuron population and its subsequent reconstruction from the neuron responses (see the upper part of **Figure 2**). In an abridged form, the theory is the following (Eliasmith and Anderson, 2004). A neuron population with a transfer function *G* is instantiated:

(1) $$a_{i}=G\left(\alpha_{i}\cdot i_{syn,i}+b_{i}\right),$$

with *i*_*syn,i*_ as input current, α_*i*_ as gain factor and *b_i_* as offset. The transfer function can be e.g., that of a Leaky-Integrate-and-Fire neuron (LIAF), building a spike rate response *a*_*i*_ from *i*_*syn,i*_. A vector variable $\vec{\text{x}}$ is then encoded in this synaptic current:

(2) $$i_{syn,i}=\vec{{\text{e}}_{i}}\cdot \vec{\text{x}}.$$

**Figure 1:**
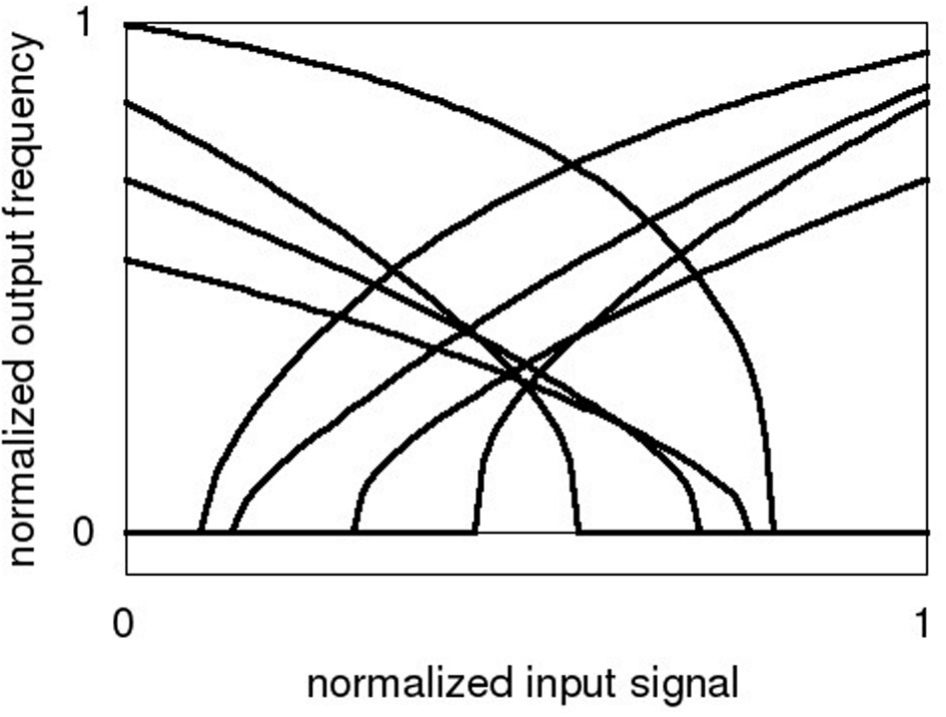
**Sample tuning curves for neurons in the NEF framework, normalized input signal $\vec{\text{x}}$ and normalized output frequency *a*_*i*_**.

The encoding vector $\vec{{\text{e}}_{i}}$ can be thought of as the preferred direction vector for that neuron: the vector for which that neuron will fire most strongly. To project the input vector into a sufficiently high-dimensional representation, α_*i*_ and *b*_*i*_ are varied between individual neurons. At the same time, allowing this variance in the neuron parameters enables a simple encoding vector composed of only discrete values. Usually, a binary vector consisting of +1 and -1 is chosen (Eliasmith and Anderson, 2004). Example tuning curves of neurons (*G*(α_*i*_ · *i*_*syn,i*_ + *b*_*i*_)) are shown schematically in Figure 1.

While Equations 1 and 2 allow us to convert a vector $\vec{\text{x}}$ into neural activity *a*_*i*_, it is also important to go the other way around. That is, given some neural activity, what value is represented? The simplest method is to find a linear decoder with decoder vector $\vec{\text{d}}$_*i*_. This is a set of weights that maps the activity back into an estimate of $\vec{\text{x}}$, as follows:

(3) $$\hat{\vec{\text{x}}}=\Sigma a_{i}\vec{{\text{d}}_{i}}.$$

For this, the neuron tuning curves are characterized across the input space $\vec{\text{x}}$. This is usually done in a regular raster. Specifically, for the scalar input x of the NEF ADC, 50 sample points spaced linearily across the normalized input range are applied as DC levels of 1 s duration and the neuron output rate measured. Given these characterized tuning curves, the optimal decoder weights for reconstructing $\vec{\text{x}}$ can be computed (Eliasmith and Anderson, 2004):

(4) $$\vec{\text{d}}=\Gamma^{-1}ϒ\;\Gamma_{ij}=\Sigma_{x}a_{i}a_{j}\;{ϒ}_{j}=\Sigma_{x}a_{j}\vec{\text{x}}$$

The sum over x denotes the sum over the single characterization points of the tuning curves. The above matrix operation arrives at the least mean squared error fit for the decoder weights for a given transformation, as was also demonstrated in Mayr et al. (2008) for spectral reconstruction of a pixel sensor array (Henker et al., 2007). Please note that the decoder weight computation is given in Equation 4 for a linear reconstruction, but various non-linear transformations of $\vec{\text{x}}$ are also possible (Eliasmith and Anderson, 2004). The decoder weights $\vec{\text{d}}$ and an exponential postsynaptic current (PSC) kernel are then applied to each spike *n* of neuron *i* to arrive at the decoded signal $\hat{\vec{\text{x}}}$:

(5) $$\begin{array}{l} \;\hat{\vec{\text{x}}}=\sum_{i}\left\{\sum_{n}\left[h_{i}(t-t_{n})\right]\vec{{\text{d}}_{i}}\right\}\\ \text{with }h(t)=\frac{1}{\tau_{psc}}\cdot \Theta (t)\cdot {\text{e}}^{-\frac{t}{\tau_{psc}}}, \end{array}$$

where Θ(*t*) is the step function. This theory can be extended to multiple networks and to symbol manipulation (Eliasmith and Anderson, 2004), but for our purposes, encoding a signal and decoding a transformation of that signal are sufficient.

### 2.2. An analog-digital-converter based on the NEF

**Figure 2:**
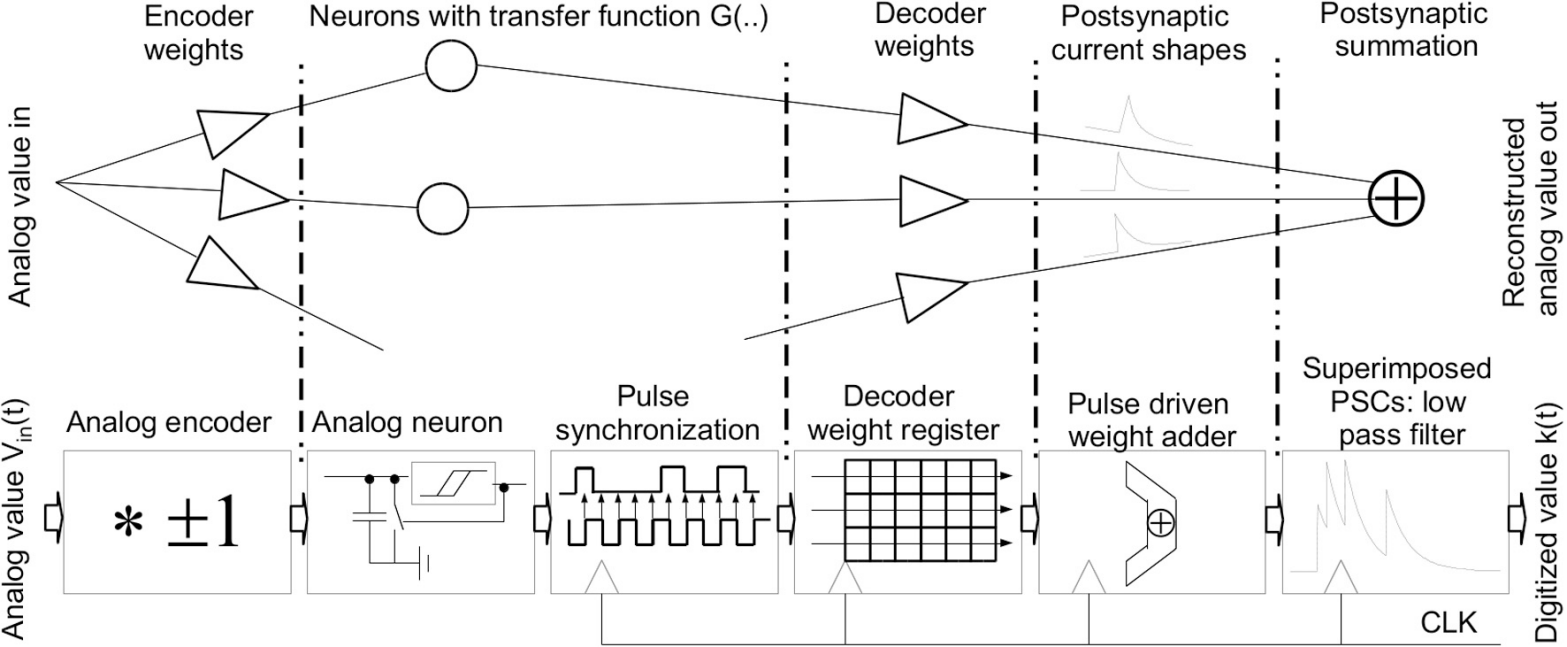
**Basic principle of NEF (upper diagram): encoding of an analog value, neuronal representation, decoding and signal reconstruction through an overlay of PSCs; Using NEF as an ADC (lower diagram): analog encoder and neuron representation, neuron pulse synchronization and digital decoder and lastly signal reconstruction via the PSC kernel**.

The basic concept of using NEF as a single-channel ADC is shown in the lower part of Figure 2. The input vector $\vec{\text{x}}$ of Equation 2 is collapsed to a single scalar value V_*in*_(t). The initial step is to build a set of analog neurons that have varying tuning curves in both encoding directions (Equation 1). Then, these tuning curves are characterized and a set of decoding weights for a linear representation is computed (Equation 4). In operation, the analog input signal is applied to all neurons in parallel. The neurons feed their spikes into a synchronizer and a subsequent clocked digital decoder that operates on digitized versions of the decoder weights. An accumulator tree summarizes all spike contributions for a given clock cycle. Please note: Since the single PSC trains employed in Equation 5 are superimposed linearly and the exponential function is self-similar, the order of this computation can be commuted. Thus, in the NEF ADC, the exponential kernel is applied on the weighted spike summation as computed for each time step, thus simplifying the digital processing. This also eliminates the need for dedicated analog PSC circuits (Noack et al., 2011). The output k(t) of this exponentially decaying sum is the digital transformation of the analog value, i.e., the ADC output.

In essence, the transfer characteristic of the ADC is built up from the single neuron tuning curves via the decoder weights. Thus, the decoder parametrization gives the transfer characteristic of the ADC. Afterwards, a low-pass filter is applied through the PSCs to suppress the high-frequency components caused by the neuron pulses.

### 2.3. Performance measures for ADCs

To characterize the performance of the NEF ADC, comparison measures with conventional ADCs are required. The main characteristics of an ADC are its resolution (number of bits in each digital output word corresponding to an analog input sample), its sample rate (number of digital words representing analog values per second), its response to a DC step at the input and its conversion latency (i.e., time from analog input to digital conversion). As the NEF ADC does not follow a conventional ADC processing chain, these are not obvious in the current context. In section 3.1, we will derive analytical and empirical couterparts for these characteristics for the NEF ADC.

Besides these baseline characteristics, there are a number of performance figures that are usually employed to estimate the performance of an ADC. The effective number of bits (ENOB) is a measure where an analog DC signal is applied to the input and the sigma of the resulting histogram of output codes is computed (Baker, 2008). In essence, the ENOB computes the limit of the ADC resolution, that is the level where the output code moves from being correlated with the input signal toward noise. As the ENOB is a single DC level measure, it provides no information about the linearity of the transfer curve.

The transfer curve can be characterized by the integral nonlinearity (INL). For the INL, a ramp is applied to the input and the deviation of the overall transfer characteristic from the ideal one is computed (Provost and Sanchez-Sinencio, 2003). The maximum INL as a scalar measure provides information about the linearity limit of the ADC. As we will show later, a plot of the INL across the input DC level is also informative, as it shows the causes of the INL limits in terms of the NEF ADC design parameters.

The signal to noise-plus-distortion (SINAD) ratio uses a sine signal at the input, to measure the amplitude of the signal in the digital output minus the harmonics and noise (Baker, 2008). In this work, we have chosen INL and ENOB as the main performance indicators, as they capture a large part of the overall ADC characteristics and are easiest to simulate and extract.

### 2.4. Overall circuit design of the NEF ADC

The circuit design for the NEF ADC was carried out in a digital 180nm CMOS process, with a VDD of 1.8 V (digital and analog). The main goal of the circuit design is to transfer as much functionality into the digital domain as possible. Therefore, only the neurons are designed in analog circuitry, while decoder, adder and exponential decay are done digitally. The second goal is to incorporate a significant amount of runtime configurability. Therefore, the decoder weights, the PSC time constant and the sample rate are configurable. The size of the neuron population employed can also be adjusted by disabling some of the decoder weights.

### 2.5 The neuron circuit

**Figure 3:**
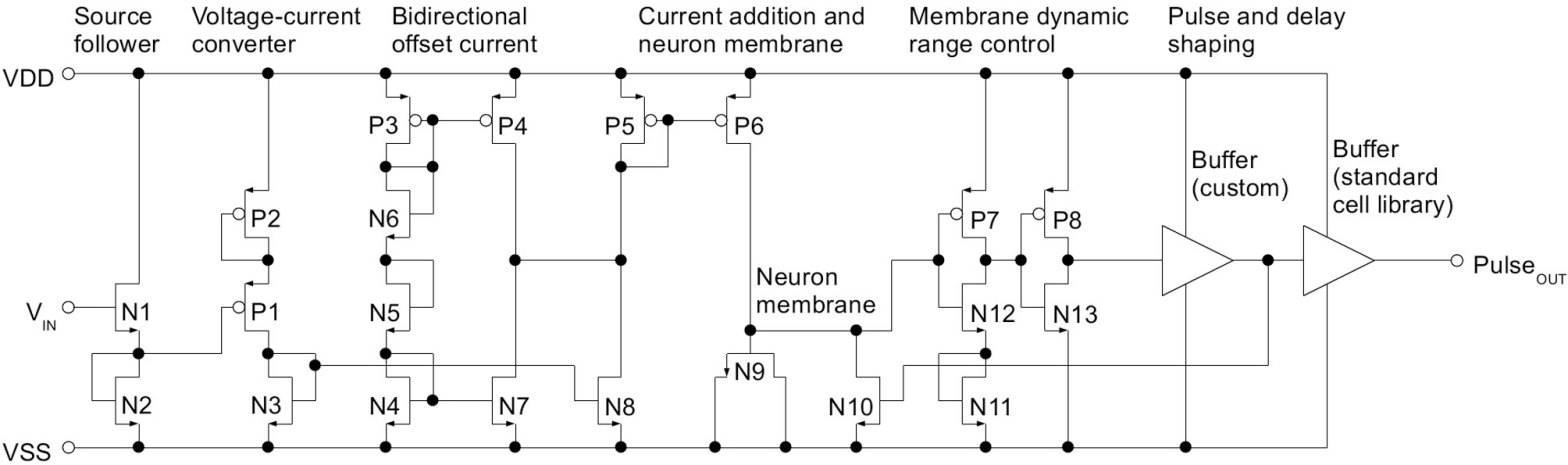
**Schematic of the analog neuron, type negative**. For clarity, powerdown switching transistors have been omitted

The overall goal of the neuron circuit development is actually quite non-intutive: The transfer curves and therefore all analog parameters have to vary as much as possible to achieve a good coverage of the input dynamic range (Eliasmith and Anderson, 2004). The parameter variance introduced by manufacturing the IC in silicon, which is generally a detrimental effect, can be employed advantageously there. Since the NEF does not place specific demands on the qualitative neuron characteristic, basic Integrate-and-Fire (IAF) neurons were chosen. The voltage input signal (see Figure 2) is applied to all neurons in parallel. The binary encoder of Equation 1 is realized by having two different types of neurons, one with a positive tuning curve and one with a negative curve (see Figure 1). Figure 3 shows the schematic of the negative neuron, i.e., the neuron type where an increase in input voltage results in a lowering of the output frequency.

The neuron operates in asynchronous mode, with its pulse frequency determined solely by the input signal. At the left of the circuit, the signal enters at the gate of a source follower N1 and its current source N2. Both N1 and N2 are high threshold voltage types to reduce cross current. Through the V_*th*_ shift, the source follower extends the dynamic range of the subsequent voltage-current converter. The input range at N1 is rail to rail, i.e., GND to 1.8 V. Transistor P1 converts the offset input voltage to a current, with the source degeneration of P2 acting as a virtual increase of the gate length of P1. Again, P1 and P2 are high threshold voltage types. Thus, P1 and P2 can both be minimum sized for increased variation and still not draw excessive cross current. N4 through N6 and P3 (all minimum sized) generate a biasing current that is subtracted from itself via P4 and N7. This generates a bidirectional mismatch current which at N8 is added to the current caused by the input signal. The resulting current is mirrored via the (minimum sized) current mirror P5/P6 on the neuron membrane capacitance N9. N11, N12 and P7 form the inverter that defines the voltage threshold for neuron firing. As with P1, N12 is source degenerated to achieve a large virtual gate length which sets the threshold high and thus extends the dynamic range of the neuron membrane. Beneficially, this also decreases the cross current despite using minimum sized transistors. P8 and N13 delay and buffer the resulting pulse signal. A subsequent custom buffer stage further increases delay before the pulse resets the neuron membrane via N10.

A standard cell buffer shapes the pulse edges for output to the synchronuous digital part. In contrast to the variation-optimized analog part of the circuit, this pulse generation is designed in a more conventional way with minimized deviations. This stems from the fact that the pulse output signal has to conform to the timing specifications of the synchronous digital part (especially, a minimum low time between pulses and a minimum pulse length).

In terms of cross current and power, voltage biasing may have been the better choice for e.g., N2, P2 or N11. However, a design choice was to make the cell self-biased in preparation for inclusion in a digital cell library flow. Thus, the only analog input is the voltage to be digitized.

In terms of the tuning curve, mismatch at the current offset gives variation along the y-axis (compare Figure 1), while V_*th*_ deviation in the source follower results in x offset. The multiple current mirrors the signal is copied across and the gain deviation of P1 result in variation of the tuning curve slope.

Although all parts of the neuron operate above threshold of the CMOS transistors, subthreshold operation as in more conventional neuromorphic circuits (Bartolozzi and Indiveri, 2007) would actually be beneficial in this application, as variations are significantly higher than in above-threshold operation (Pineda de Gyvez and Tuinhout, 2004; Giulioni et al., 2012). However, only very slow ADCs would be possible this way (Yang and Sarpeshkar, 2006).

**Figure 4:**
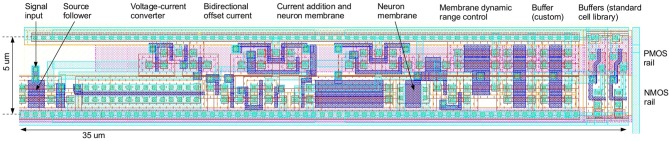
**Layout of the negative type neuron in UMC 180 nm CMOS technology, with signal flow from left to right similar to the schematic in Figure 3**.

Figure 4 shows the layout of the neuron of Figure 3. The signal flow from left to right is similar to the schematic. No attention was given to matched layouting or similar, as variation should be maximized. The layout has been optimized for an eventual integration in a digital standard cell flow. Only one metal layer is used and the cell is compatible with the 5 μm Faraday digital standard cell raster.

### 2.6. The decoder and PSC summation

**Figure 5:**
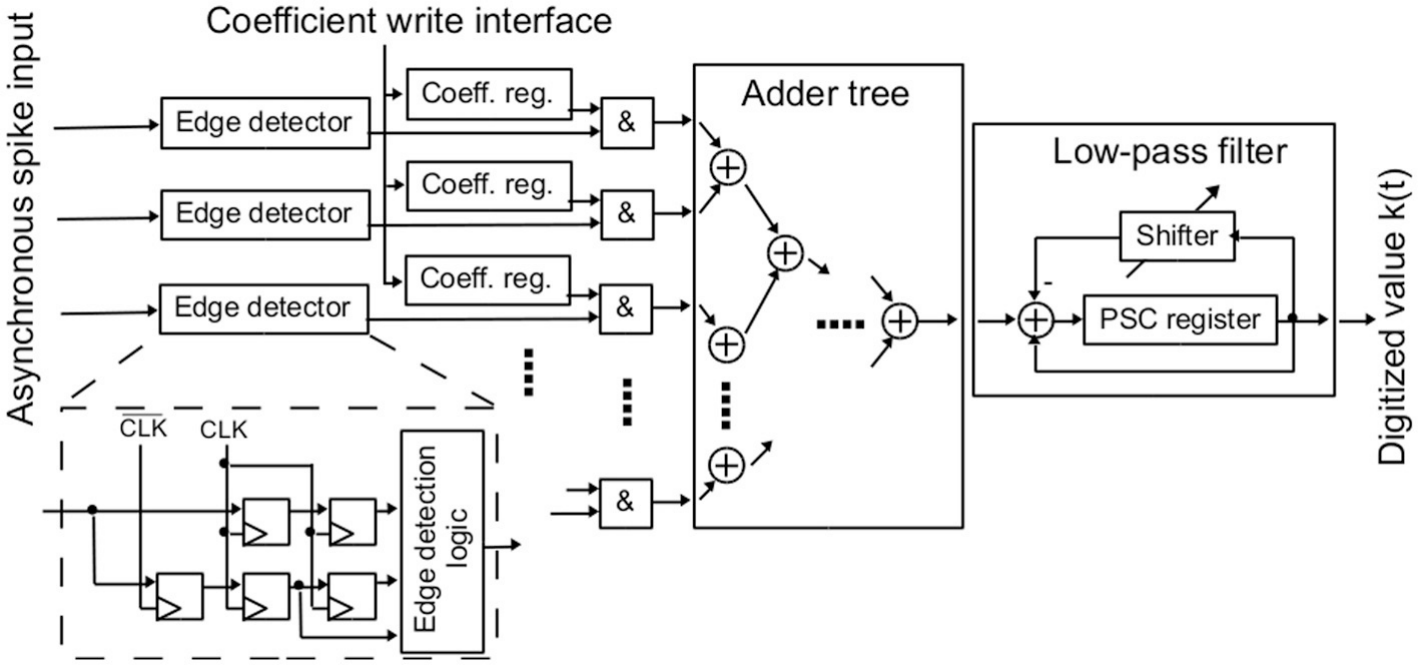
**Digital synchronous pulse registration, adder tree and low-pass filter**.

The digital part of the NEF ADC is shown in Figure 5. It consists of synchronizer circuits for the input pulses of the analog neurons, decoder weight registers, an adder tree for summing all active decoder weights, and a low-pass filter.

The asynchronous pulses of the analog neurons have to be synchronized to the digital clock. Furthermore, the synchronized output should be independent of the neuron's pulse length. For this, a rising edge detector is used, which generates an output signal that is high for exactly one clock per pulse. For achieving full throughput, i.e., being able to detect one spike per clock cycle, the asynchronous pulse signal is sampled at both positive and negative clock edge via two standard two-stage synchronizers. An additional register stores the signal level at the previous negative clock edge. With this structure, a sequence negedge-posedge-negedge is available at the synchronizer output. An edge detection logic detects low-to-high changes in the input signal from this sequence.

The above edge detector captures all pulses in the asynchronous input signal, as long as the pulse length and the time the input is low between spikes is each higher than half a clock period. If the pulse length is shorter than this, only a fraction of the spikes is detected, attenuating the neuron's transfer function by a factor. In principle, the same happens if the input signal is low for a too short time between spikes. As this low time is decreasing with the pulse rate, the neuron's transfer function would start decreasing at high rates. While not intended, both effects would still be covered by the calibration of the NEF ADC.

As shown in Figure 5, each synchronized spike output activates its individual decoder weight register. The values of these registers are written via a separate configuration interface. The decoder weights effectively allow for setting up the transfer function of the AD conversion. The bit width of the decoder weights is a crucial parameter. The weight registers consume a significant part of the whole circuit area, so the bit width should be as small as possible. However, a certain minimum bit width is needed to not limit resolution of the AD conversion. In the current design, 8 bit signed values were used for achieving sufficient flexibility.

An adder tree calculates the sum over all active decoder weights. It was designed as a pipelined structure to achieve a throughput of one adder tree result per clock cycle. Computing across all spikes in a parallel manner as in the adder tree also obviates the need for any spike sorting or arbitration that would otherwise be required (Scholze et al., 2010)

The adder tree results are fed into the low-pass filter, resembling the PSC signal reconstruction. The low-pass filter result constitutes the output of the AD conversion. In each clock cycle, the current output of the adder tree is added to the low-pass filter's PSC register. At the same time, the current PSC register content is shifted right and subtracted, resulting in the desired first-order low-pass characteristic. The shift width *b* is configurable. The resulting PSC time constant τ_*psc*_ can be derived from the clock frequency *f*_clk_ and *b* by equating the result of the shift operation with an exponential decay:

(6) $$\text{PSC}(\text{t})\cdot {\text{e}}^{-\frac{1}{f_{\text{clk}}\cdot \tau_{psc}}}=\text{PSC}(\text{t})\cdot (1-2^{-b}).$$

Applying the first order Taylor series approximation for small exponents to the left hand side of Equation 6, the following expression is derived

(7) $$\tau_{psc}=\frac{2^{b}}{f_{\text{clk}}}.$$

As can be seen from Equation 7, realizing the PSCs in digital allows setting arbitrarily long time constants, which are necessary for a high-resolution ADC. Achieving the same in analog circuits would be difficult, especially in deep-submicron technologies (Noack et al., 2012).

The digital part of the NEF ADC was described in Verilog to be completely compliant with the standard digital design and synthesis flow. Thus, it can be easily ported between technologies.

## 3. Results

The following two sections contain results of the NEF ADC based on neuron models simulated in Nengo and Spice simulations of the actual transistor-level neuron circuits. For quick reference, we give in Table 1 the baseline ADC characteristics we use in both cases.

**Table 1:** **Baseline characteristic of NEF ADC for Nengo and circuit level hardware simulations**.

**ADC**	**Baseline**	
**characteristic**	**Nengo**	**Hardware**
τ_*PSC*_	128 ms	853 ns
*N_neuron_*	512	512
*W_res_*	8 bit	8 bit
*f_neuron,max_*	400 Hz	45 MHz
*T_synch_*	1 ms	6.67 ns
Input range	idealized 0..1	GND to 1.8 V
Tuning curve	set by	intrinsic through
spread	parameters	transistor mismatch

The baseline characteristics of the Nengo simulations are: τ_*PSC*_ = 128 ms (i.e., a shift of 7 bit, compare Equation 7), decoder weight resolution *W*_*res*_ = 8 bit (compare section 2.6), maximum rate of the IAF neurons *f*_*neuron,max*_ = 400 Hz, and a population of *N*_*neuron*_ = 512. The spike times from the Nengo simulation are synchronized to the clock of the digital system model *f*_clk_ = 1 kHz, i.e., the resolution of the pulse registration in the baseline is *T*_*synch*_ = 1 ms.

**Figure 6:**
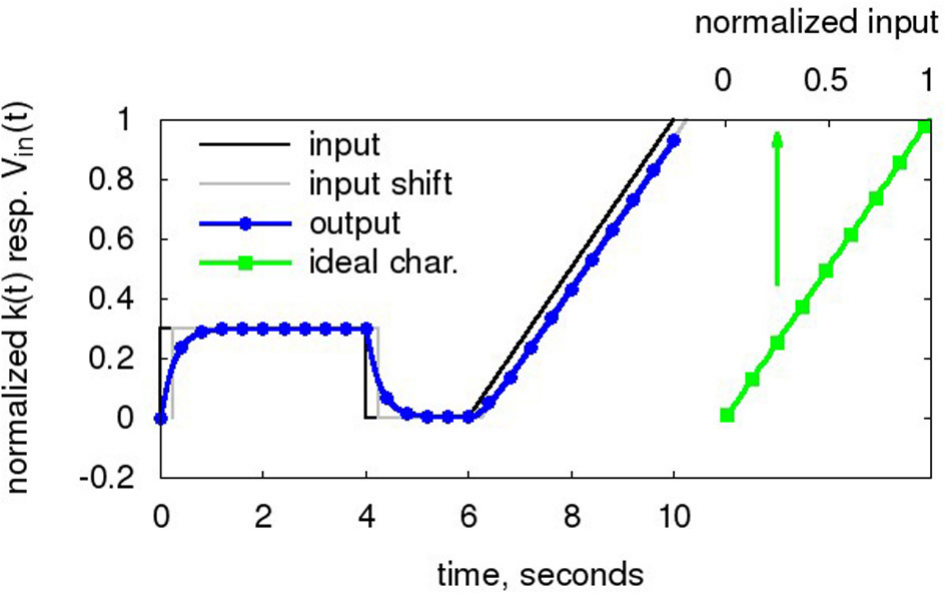
**Input waveform V_*in*_(t) (black)**. The Nengo simulator takes this normalized waveform as input. The digitized output k(t) (blue, circles), i.e., the state of the low-pass filter, is also normalized to 0..1. For comparison to the output, V_*in*_(t) shifted by τ_*PSC*_ is also displayed (black, dashed). Also shown is the ideal transfer characteristic (green, dashed, squares) as computed from the decoder weights and tuning curves in Equation 3. Baseline taken from Table 1, with τ_*PSC*_ of 256 ms for enhanced delay visibility.

The baseline for the transistor-level simulations is the same as for the Nengo neurons, with the following modifications: The VDD of the neurons is 1.8 V, i.e., the normalized input signal V_*in*_(t) of Figure 6 is mapped to a voltage swing of 0..1.8 V. The system model of the digital building blocks is sped up from the 1 kHz clock to 150 MHz, i.e., *T*_*synch*_ = 6.67 ns, to be compatible with the hardware neuron speed. The PSC time constant is adjusted by the same factor, i.e., a τ_*psc,biol*_ = 128 ms in the Nengo simulations is equivalent to τ_*psc,tech*_ = 853 ns in the transistor-level simulations. However, for comparison of the results of section 3.2 with section 3.1, the timebase is converted back to 1 kHz for all data plots except **Figure 10**.

A note on simulated time vs. execution time: The simulated time is the reference time of the simulation, which is biological real time in Nengo, i.e., if the PSC time constant is set to 30 ms, the PSC decays 63% in 30 ms simulated time. On the other hand, as CMOS circuit frequencies are inherently much higher, simulated time and all time constants can be chosen much shorter for these simulations. In actual hardware, this has the beneficial effect of increasing the conversion speed of the NEF ADC. On the other hand, execution time means the time it takes to run a certain input waveform on the network in either Nengo or Spice. The execution time is significantly less in Nengo as the simulator is optimized for neuron models and the neurons are abstracted to a set of equations. In contrast, the spice simulations deal with the transistor-level neurons and incorporate parasitic capacitances and resistors, which makes them significantly slower to execute.

A system model of the digital building blocks outlined in section 2.6 is used for the processing of the neuron output spikes of both the Nengo as well as the transistor level simulations. The system model has been verified against the synthesized Verilog code. The decoder weights are computed for a linear ADC characteristic for easy comparison with conventional ADCs.

### 3.1. Results of an ideal implementation

To evaluate the efficacy of using the NEF framework as an ADC without carrying out analog hardware design, the initial implementation was done in the Nengo simulator (Stewart et al., 2009) with idealized neurons, having controlled tuning curve spread. IAF neurons are employed for compatibility to the hardware neurons, but there are negligible differences in results to e.g., LIAF neurons. Apart from allowing large parameter sweeps due to the reduced simulation time, using ideal simulated neurons also helps to establish a baseline performance that can be compared to the hardware neurons.

As can be seen in Figure 6, the waveform entered in the NEF ADC consist of an initial DC level for ENOB computation (0 to 4 s), DC level at 0 for ADC settling a the lower input limit (4 to 6 s) and a subsequent ramp for INL computation (6 to 10 s). The ramp is deliberately slow so that sections of it can be used as collection of quasi-DC levels at different input voltages to characterize the tuning curves of the neurons. The decoder weight vector is computed according to Equation 4 based on 50 input level sample points. Two important characteristics can already be observed: The digitized output has an exponential step response settling with τ_*PSC*_. Also, the digitized output lags the input by τ_*PSC*_ at the input ramp, constituting the ADC latency.

**Figure 7:**
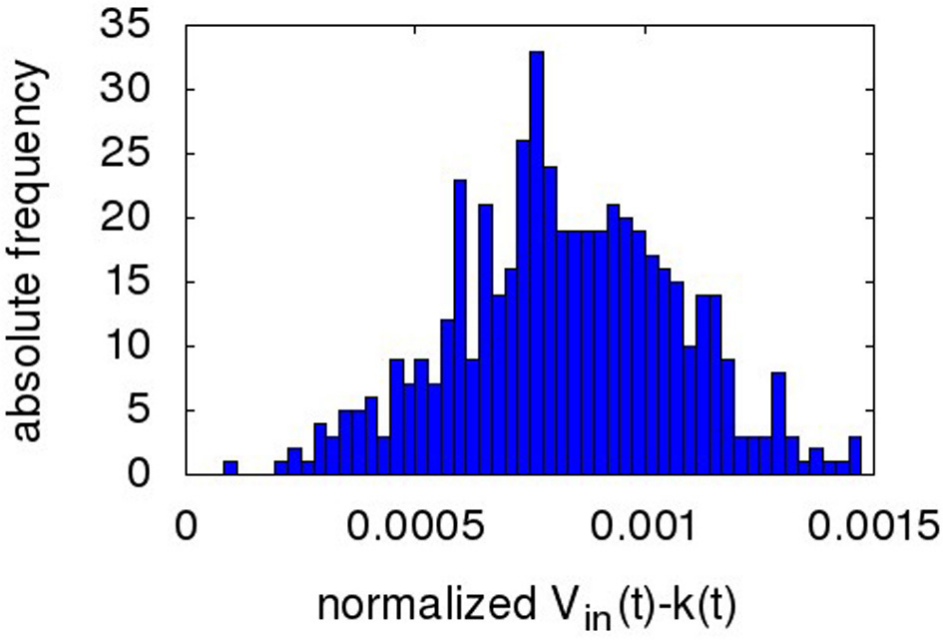
**Histogram of the digitized NEF output minus ideal analog input (both normalized to a dynamic range 0..1) during the time slice 2.9 to 3.4 s of the output wave in Figure 6 (i.e., the settled portion of the first DC input)**.

Figure 7 shows a sample ENOB plot. The digitized output k(t) in the timespan from 2.9 to 3.4 s is subtracted from V_*in*_(t) and the difference plotted in a histogram. The ENOB is given by the standard deviation of this distribution (Baker, 2008). As can be seen, the NEF ADC output is similar to a conventional ADC, i.e., a DC level is replicated in the form of a narrow distribution of output codes around it. Despite the pulsing nature of the overall network and the high spread in decoder weight values (>20 max/min weight, i.e., a high amplification of some spike trains compared to others), there are no corresponding large transients in k(t).

**Figure 8:**
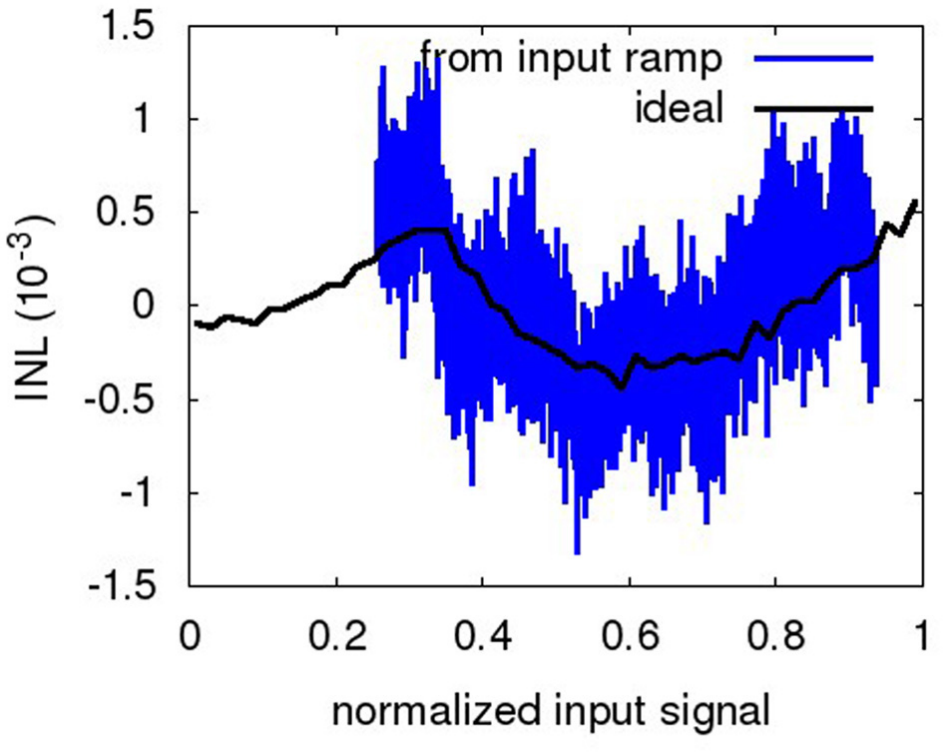
**INL of ramp portion of the waveform in Figure 6 (relative to the normalized full swing)**. The ideal INL based on the transfer curve of Equation 3 is also displayed (black, dashed).

Figure 8 shows a sample INL curve based on the ramp portion of the input waveform. For the low input voltages, the initial INL exhibits a residue from the settling to the ramp at *t* = 6 s. This is discounted for in the INL computation. The INL given in the following is the ±max deviation from the ideal curve (Provost and Sanchez-Sinencio, 2003), with respect to the normalized input range. The INL curve is not as characteristic as that of a more conventional ADC (Chae et al., 2013), as the transfer curve of the NEF ADC is built in a random fashion by the decoder weight computation based on the individual neuron deviations. The curve shown is representative for the NEF ADC, i.e., the INL curves are smooth but exhibit no characteristic shape. The ideal INL curve based on the transfer curve as computed from the decoder weights and tuning curves (compare Figure 6) is also shown. It can be observed that they match reasonably well, with the dynamic, ramp-based INL exhibiting additional high-frequency noise due to the network pulse activity. Increasing τ_*PSC*_ dampens the noise on the dynamic INL, reducing it to the level exhibited by the ideal INL. However, the ideal INL constitutes the lower bound, as it is determined largely by the number of neurons and thus is static and not amenable to further filtering.

The maximum INL for each datapoint shows a very steady 2 bit difference to the ENOB, e.g., the baseline example with ENOB 10.98 bit has a maximum INL of 8.91 bit. Unless otherwise noted, we will thus employ mainly ENOB as ADC performance characteristic, as it is more easily computed. Table 2 details the behavior of the ENOB for a sweep of every variable given in the baseline description above. While some of these scaling characteristics of signal representation with network parameters have been explored for NEF (Choudhary et al., 2012), a full sweep of all relevant parameters has not been shown so far.

**Table 2:** **Scaling of ENOB with design characteristic/variable**.

**ADC characteristic**	**Example**		**ENOB scaling with characteristic**	**Remark**
	**1**	**2**		
_τ*PSC*_	8.98 bit|_τ_*PSC*_=32 *ms*_	9.99 bit|_τ_*PSC*_=64 *ms*_	linear	
*N_neuron_*	8.16 bit|_*N_neuron_*=32_	9.65 bit|_*N_neuron_*=128_	*ca*. $\sqrt[{1.5}]{\;}$	
*W_res_*	11.00 bit|_*W_res_*=5 *bit*_	10.92 bit|_*W_res_*=3 *bit*_	no dependence	See hardware discussion, has to be above a lower bound
*f_neuron,max_*	7.69 bit|_*f_neuron,max_*=50 *Hz*_	9.73 bit|_*f_neuron,max_*=200 *Hz*_	linear	Scaling saturates at approx. 0.5· *T_synch_*
*T*_*synch*_	6.81 bit|_*T_synch_*=2 *ms*_	5.90 bit|_*T_synch_*=4 ms_	linear	Please note: For this sweep, *f*_*neuron,max*_ = 50 Hz to avoid saturation

Baseline characteristic as in Table 1, with resulting ENOB 10.98 bit.

The scaling behavior of the ENOB can be extracted from three data points for each variable (baseline, example 1 and 2). Not surprisingly, there is linear scaling of ENOB with τ_*PSC*_, *f*_*neuron,max*_, and *T*_*synch*_. All three variables affect the number of neuron pulses that are taken into account for a single output code to average over. This can be thought of as similar to scaling of resolution with the oversampling ratio (OSR) in a conventional first- order DSM (Perez et al., 2011). There is a saturation of ENOB with *f*_*neuron,max*_ at about half the frequency given by *T*_*synch*_ (data not shown). This could be due to a saturation of the pulses per timestep, i.e., if there is on average more than one pulse per two timesteps, not much additional information is conveyed.

**Figure 9:**
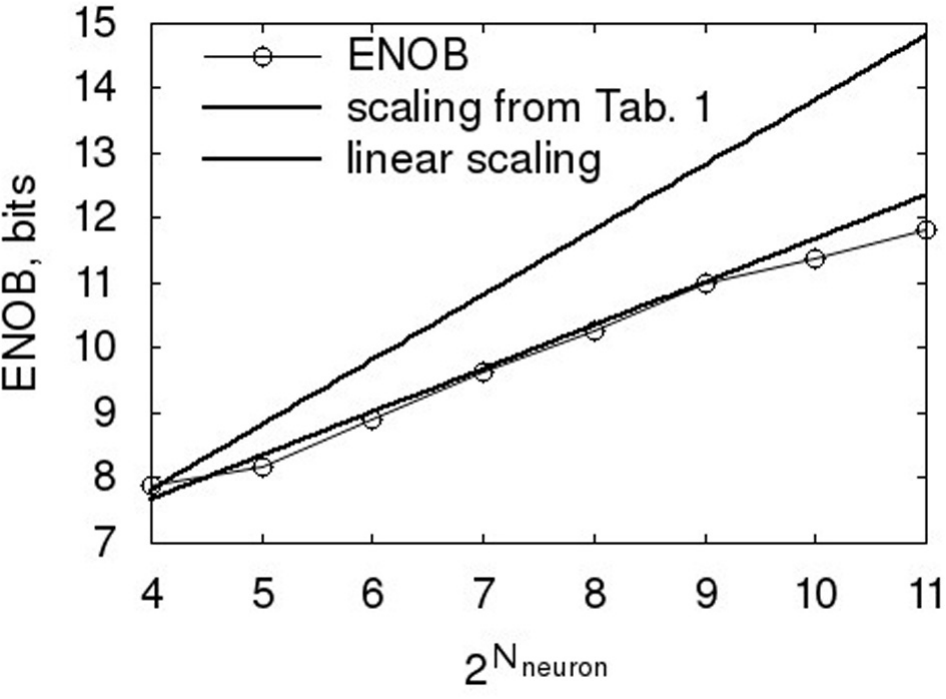
**Scaling of ENOB with the number of neurons**. Baseline characteristic as in Table 1.

The ENOB scaling with *N*_*neuron*_ is worse than the scaling above for τ_*PSC*_, as shown in Figure 9. So the naive assumption, that an increase in *N*_*neuron*_ results in a proportional increase of pulses for a given output code and thus gives linear scaling, does not hold. At the same time, it is slightly better than the expected $\sqrt[{2}]{}$ factor resulting from applying a signal to independent ADCs (King et al., 1998). The likely cause is that the neurons cannot be thought of as independent, as the ADC transfer characteristic is built from them and thus the decoder extracts the best fit transfer based on a combination of all of them.

The scaling of *W*_*res*_ also relates to the construction of the transfer characteristic: Surprisingly, there is almost no dependence between *W*_*res*_ and ENOB, i.e., the decoder weight can be quantized quite severly after computation and still result in a high-fidelity k(t). Intuitively, if the decoder weights have access to a widely varying neuron population, their own variation can be very limited. There is only an empirical lower bound of *W*_*res*_ that has to be fulfilled to achieve reconstruction of V_*in*_(t) at all. This detail will be revisited in section 3.2.

The equivalent sample rate and Nyquist frequency are still missing from this characterization of the NEF ADC. The Nyquist signal frequency can be derived from the slew rate of a sine input signal, based on the assumption that it is reconstructed via τ_*PSC*_ with an exponentially decaying kernel. The sine has a maximum downward slew rate (at *t* = 1/(2*f*)), which can be equated to an exponentially decaying PSC starting at *t* = 1/(2*f*) with an amplitude of 0.5:

(8) $$\frac{\text{d}\left\{0.5\cdot \text{sin}(2\pi ft)\right\}}{\text{d}t}{|}_{t=\frac{1}{2f}}=\frac{\text{d}\left\{0.5\cdot {\text{e}}^{\frac{t-\frac{1}{2f}}{\tau_{PSC}}}\right\}}{\text{d}t}{|}_{t=\frac{1}{2f}}.$$

Solving this, we receive the maximum frequency *f*_*sig,max*_ that a full-swing sine wave is supported by a given τ_*PSC*_:

(9) $$f_{sig,max}=\frac{1}{2\pi \tau_{PSC}}.$$

With a corresponding Nyquist rate of *f*_*sample*_ = 2· *f*_*sig,max*_, i.e., the frequency at which the state of the decaying accumulator is read out. Not entirely surprising, this constitutes a first order low-pass with cutoff at 1/τ_*PSC*_.

The first-order low-pass characteristic also explains the conversion latency of the NEF ADC from a linear input ramp. The equations for the low-pass output *y* and input *x*(*t*) are:

(10) $$\tau_{PSC}\frac{\text{d}y}{\text{d}t}=-y+x(t)\text{ with }x(t)=a\cdot t$$

The corresponding solution for the low-pass output is:

(11) $$y(t)=a\cdot (t-\tau_{PSC}).$$

Thus, the low-pass output lags the input by τ_*PSC*_. As can be seen from Figure 6, the other parts of the ADC processing chain do not add significantly to the conversion latency.

Table 3 sums up the results of this section, with an empirical ENOB formula based on Table 2. The ENOB formula is valid for *f*_*synch*_ ≥ 2 · *f_neuron,max_*, i.e., for the neurons firing below saturation of the pulse registration. The scaling factor *T*_*norm*_ is approximately 0.6 ms. The dynamic range is a function of the neuron tuning curve variation, i.e., if the neurons have positive and negative responses that vary significantly even near the rails, the input can be rail-to-rail (compare Figure 1 and Figure 6).

**Table 3:** **Characteristics of NEF used as a linear ADC**.

Conversion rate	$\frac{1}{\tau_{PSC}\cdot \pi }$
Max. input frequency	$\frac{1}{2\cdot \tau_{PSC}\cdot \pi }$
Empirical ENOB formula	$ENOB(Bit)=\text{ld}(\tau_{PSC}\cdot \sqrt[{1.5}]{N_{neuron}}\cdot f_{neuron,max}\cdot \frac{1}{T_{reg,Pulse}}\cdot T_{norm})$
Conversion latency	τ_*PSC*_
Settling time to a step response	*T*_*set*_ = τ_*PSC*_ · ENOB · ln2
Dynamic range	rail-rail

### 3.2. Results of the circuit implementation

This section expands the results obtained with the Nengo neurons to the neurons described in section 2.5. We use a neuron population that is based on Monte carlo variations of a parasitic extraction of the layout in Figure 4 and its counterpart for the positive neuron.

**Figure 10:**
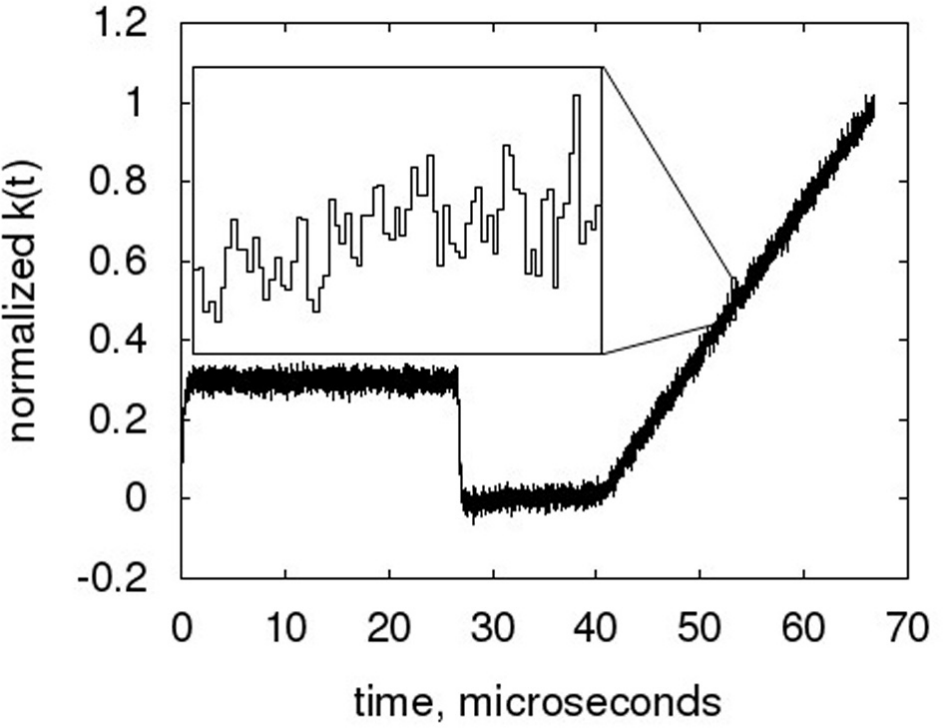
**Sample time course of the low-pass filter, with zoom of the ramp part of the waveform**. The NEF ADC is configured to the hardware baseline characteristic, but with only 128 neurons and τ_*PSC*_ = 107 ns (equiv. 16 ms) to reduce resolution and thus enhance the visibility of the curve progression from output code to output code.

Figure 10 shows the time course of the decaying accumulator at its hardware timescale. From the zoom plot in the Figure, the single code values can clearly be seen. The small time constant configured in this example allows a clear view of the fine structure of the NEF ADC output. Due to the overlay of multiple single neuron transfer curves and the dynamic nature of the neurons, this output is quite stochastic, with the τ_*PSC*_ decay not readily evident. The code transitions cannot be identified, making more conventional INL measurement difficult (Baker, 2008).

**Figure 11:**
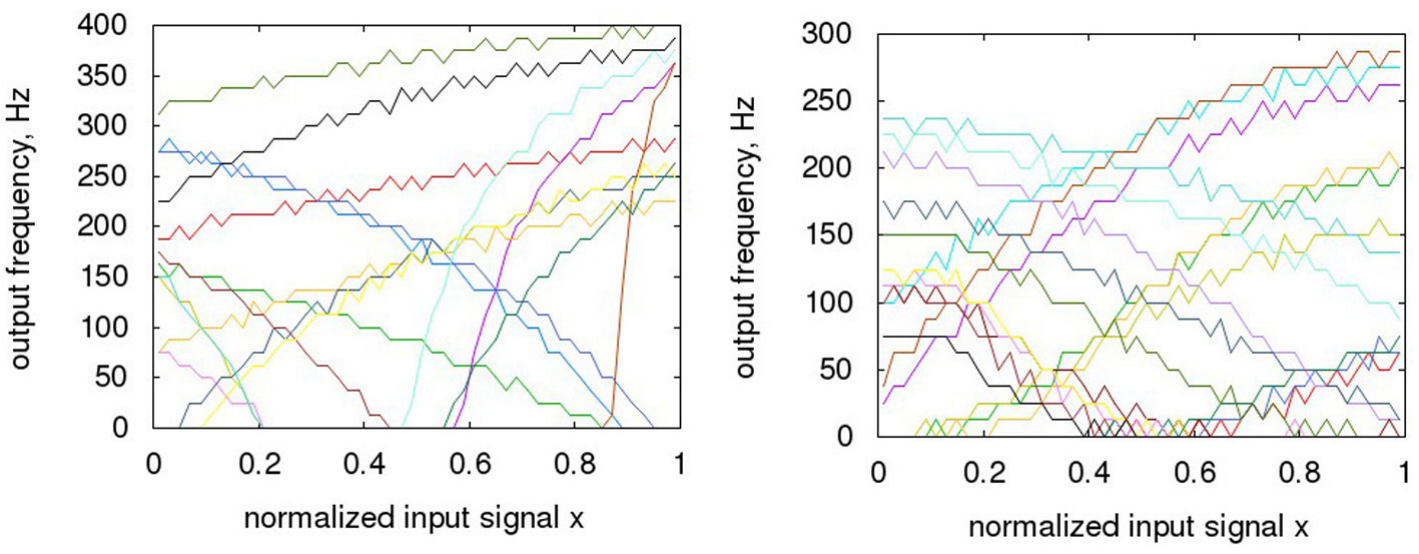
**Variation of neuron tuning curves; (A) Nengo; (B) hardware**. For the hardware Monte Carlo variations, only mismatch is activated, as expected on a single die, i.e., no process deviations. Please note: the maximum rates of the hardware neurons are actually at ca. 45 MHz, which converts to 300 Hz when the timescale is converted to the Nengo one. The normalized input range of the hardware neurons corresponds to rail-rail, i.e., GND to 1.8 V.

In Figure 11, sample tuning curves of both types of neurons are overlaid for the Nengo generated neurons and the hardware neurons. When adjusting for the time base, the hardware neurons are somewhat slower than in Nengo, but the difference is not significant, as the ENOB starts to saturate at these frequencies in any case. It can be seen that in general, the circuit measures taken in section 2.5 for the hardware neurons generate a satisfactory range of offsets in x and y direction. The complete input range is converted with sufficiently varying neuron tuning curves, with the possible exception of a range close to the two rails, as the tuning curves there tend to correlate significantly and thus resolution would drop in these areas. The Monte Carlo models were set only to mismatch (i.e., not mismatch and process) to generate this curves, so this level of spread can be expected from a large part of manufactured IC instances. However, as the spread of the curves is determined by random effects of the manufacturing process, individual instances of the ADC have to be checked for sufficient spread, thus defining a yield in terms of ADC resolution. When comparing the two families of tuning curves, the main observation is that the Nengo generated neurons tend to vary more, especially in their gain.

As can be seen from the ENOB comparison in Table 4, this has a significant impact on the overall computation. If the neurons do not encode for sufficiently different features of the input signal, the representation of the input signal degrades. Table 4 illustrates that the ENOB scaling with design characteristic is in general the same as in the Nengo simulations. However, the ENOB consistently is 1.6 bit less in the hardware. Consequently, the scaling factor *T*_*norm*_ in the empirical formula in **Table 6** is adjusted to approximately 0.2 ms.

**Table 4:** **ENOB-comparison for two examples Nengo and HW: τ_*PSC*_ sweep and *N*_*neuron*_ sweep**.

**NEF ADC parameters**		**Resulting ENOB**	
**τ_PSC_**	**N_neuron_**	**Nengo**	**Hardware**
32 ms	128	7.64 bit	6.01 bit
64 ms	128	8.65 bit	6.99 bit
64 ms	512	9.99 bit	8.29 bit
128 ms	512	11.00 bit	9.29 bit

**Figure 12:**
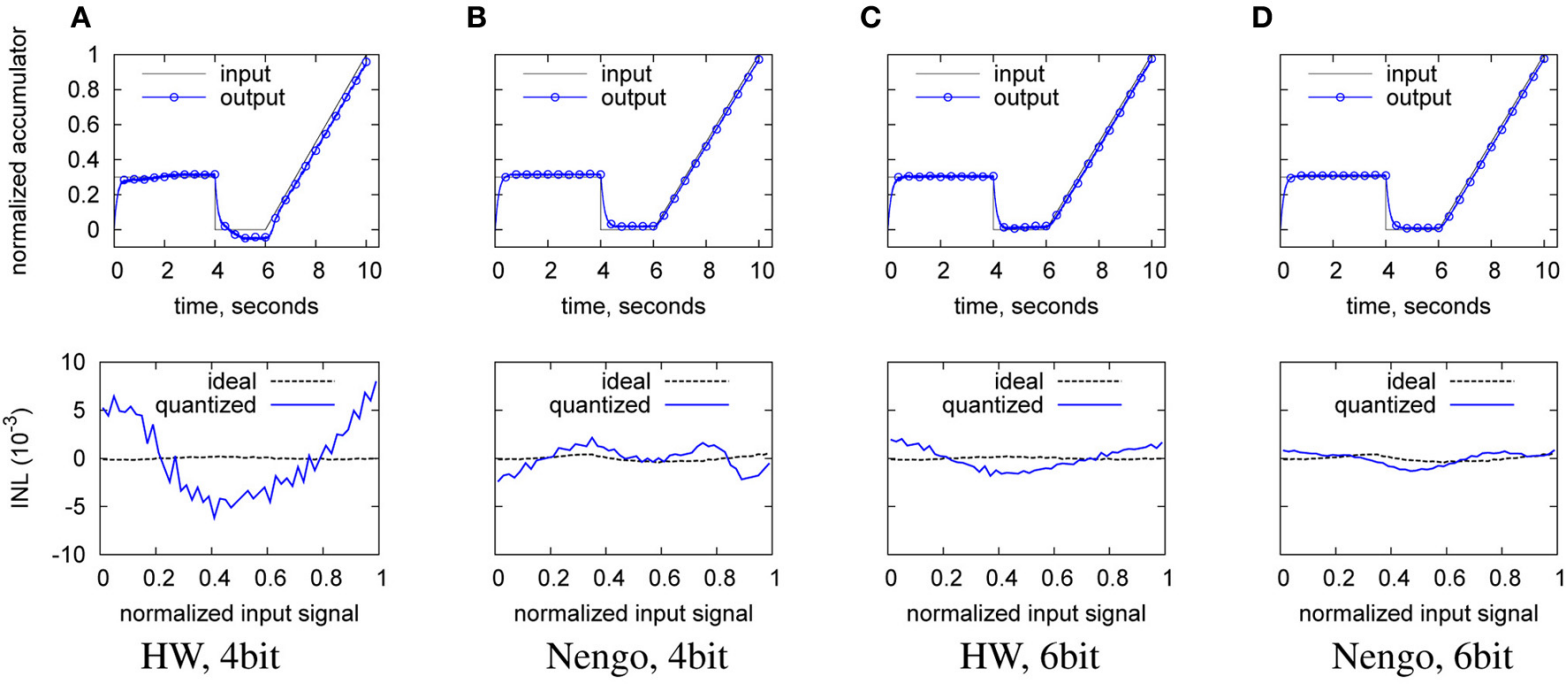
**Reproduction of the input waveform in the digitized output (top), and ideal INL (bottom), from left to right**. **(A)** with hardware neurons and 4 bit decoder weight; **(B)** with Nengo neurons and 4 bit decoder weight; **(C)** with hardware neurons and 6 bit decoder weight; **(D)** with Nengo neurons and 6 bit decoder weight. Baseline hardware characteristic, but only 128 neurons.

The reduction in tuning curve variation also has an impact on the decoder weights. Due to the lower variation in tuning curves, the network needs a higher decoder weight precision in order to replicate a given transfer characteristic (as the transfer characteristic is built out of a combination of tuning curves and weight, see Equation 5). This can be seen from Figure 12, which shows the input waveform reproduction for Nengo and hardware neurons at 4 and 6 bit decoder weight resolution. At 4 bit, severe scaling errors exist for the hardware case in the wave output compared to the input.

Table 5 illustrates the quantitative INL and ENOB repercussions. The INL trend from the lower row of the plots in Figure 12 is visible in the INL entries for 3 and 4 bit, where the hardware starts to worsen before the Nengo simulation. The INL as a measure based on a waveform better reflects this effect, the ENOB as a steady-state measure does not capture such dynamic errors sufficiently. Thus, there seems to exist a lower resolution limit for the decoder weights that is a function of the variation of the tuning curves. At 8 bit, the resolution chosen for the hardware implementation is well above this limit.

**Table 5:** **Scaling of ENOB and INL with decoder weight resolution for Nengo simulation and hardware implementation**.

	**Decoder resolution**		
	**6 bit**	**4 bit**	**3 bit**
ENOB Nengo	9.65 bit	9.60 bit	9.61 bit
INL Nengo	7.61 bit	7.63 bit	6.93 bit
ENOB Hardware	7.97 bit	7.95 bit	7.96 bit
INL Hardware	6.07 bit	5.69 bit	5.21 bit

*Baseline hardware characteristic, but with 128 neurons*.

In order to test the robustness of the analog value representation to errors in the processing chain (neurons and decoder tree), we evaluate the failure or degradation of neurons:

- Random failure of one third of overall neurons, modeled through first computing a full decoder weight set, then setting one third of decoder weights to zero. For INL and ENOB, the output signal is adjusted for the corresponding amplitude decrease.
- Random perturbation of one third of positive neurons and one third of negative neurons, modeled by first computing a full decoder weight set, then randomly permuting the decoder weights of one third of the positive and negative neurons.

The ENOB shows interesting behavior: For the perturbation case, having the neuron in the network at all, even if with a different decoder weight, leaves the ENOB at its baseline level. That is, the spikes of this neuron still contribute to a less noisy DC level because they are added and low-pass filtered. However, the ENOB is degraded (with the amount expected from the formula in Table 6) if the neurons are lost, i.e., their spikes are not counted for the digitized output value.

**Table 6:** **Consequences of neuron failures in hardware: INL and ENOB for a random failure or perturbation of one third of the neurons**.

	**Baseline**	**Neuron failure**	**Neuron perturbation**
ENOB	7.97 bit	7.64 bit	7.98 bit
INL	6.07 bit	4.83 bit	4.75 bit

*Baseline hardware characteristic, neurons reduced to 128. ENOB and INL represent the average of 10 runs with different perturbations/failures*.

**Figure 13:**
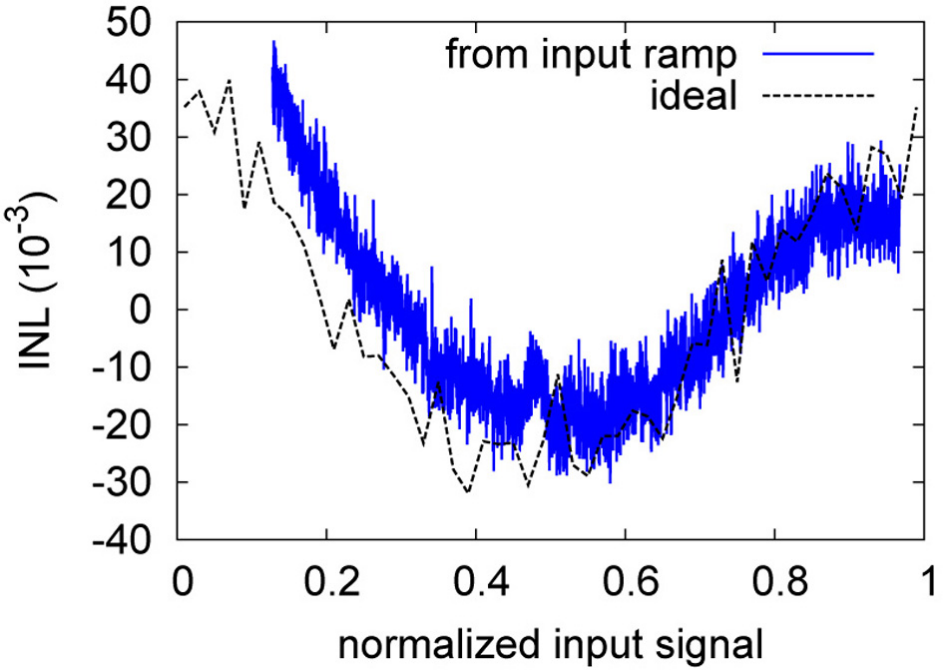
**Ideal and ramp-based INL of the neuron perturbation of Table 6**.

As for the weight resolution, the INL is the more descriptive measure. Figure 13 gives a representative example of the INL degradation due to neuron perturbation. The INL degradation for failure is similar both qualitatively and quantitatively. Table 6 shows that the INL is degraded by about 1.2 bit for both neuron failure and perturbation. Thus, not surprisingly, the transfer characteristic strongly depends on all neurons being present in the overall signal with the specific decoder weight that corresponds to their distinct manufacturing-given deviation in the tuning curve.

**Figure 14:**
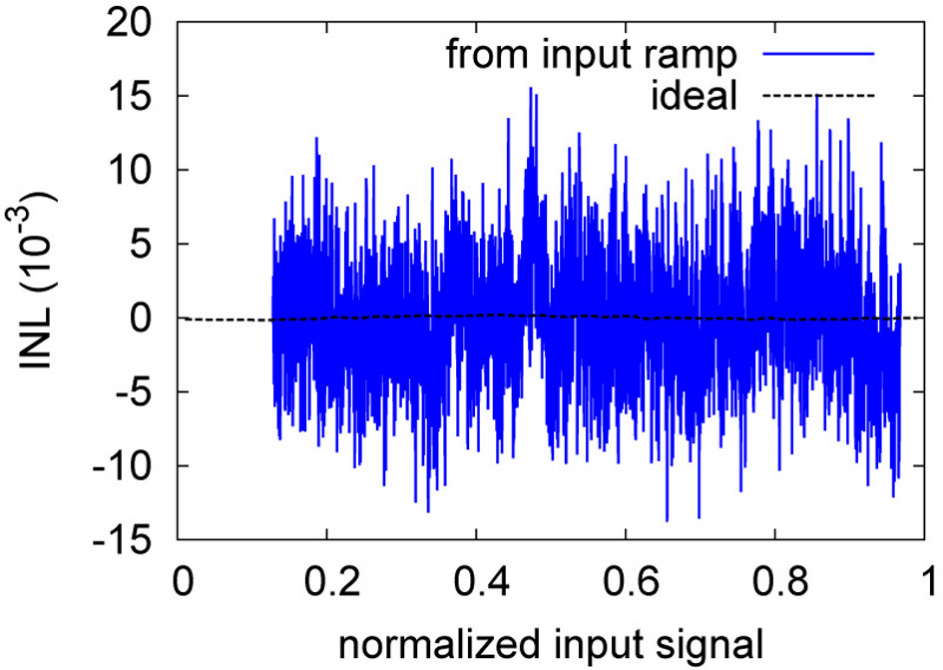
**Ideal and ramp-based INL of the baseline of Table 6**.

The INL degradation is actually more severe than evident from Table 6. As can be seen from Figure 14, the INL for the baseline is still dominated by pulse noise, while in Figure 13, static deviations clearly dominate. That is, for the baseline, a stronger low-pass-filtering could still decrease the INL, whereas for the perturbation case, further filtering does not diminish the INL.

However, judging from either Table 6 or Figure 13, the INL degradation for this quite faulty network with one third disturbed neurons/weights is still only somewhere between 1.2 and 2.5 bit. Thus, this illustrates the soft degradation properties of the overall characteristic, which is due to the distributed analog value representation across the neurons. That is, there is not a single analog block that is crucial to the overall function, in contrast to e.g., the first amplifier in a pipeline converter. In case of neuron loss as simulated above, optimized rerouting could also be used to alleviate some of the loss (Mayr et al., 2007).

In Tables 7–9, the characteristics of the NEF ADC hardware design are summed up and compared to the state of the art. The final NEF ADC design contains 1280 neurons (640 of each encoding/type), operates at f_*clk*_ = 150 MHz and a VDD of 1.8 V. The area occupied by the digital building blocks is 2.69 mm^2^, the area of the analog blocks (i.e., neurons) is 0.23 mm^2^. Its analog power draw is 40 mW, digital 120 mW. As the digital blocks are designed to be runtime configurable, three different configurations are chosen for the comparison:

- Configuration for high sample rate: 12 MSamples/s, 7.5 bit ENOB (with a shift of 2 bit, i.e., equivalent τ_*psc,biol*_ = 4 ms, actual τ_*psc,tech*_ = 26.7 ns, compare Equation 7).
- Configuration for medium sample rate: 750 kSamples/s, 11.4 bit (shift of 6 bit, τ_*psc,biol*_ = 64 ms,τ_*psc,tech*_ = 427 ns).
- Configuration for low sample rate: 730 Samples/s, 21.4 bit (shift of 16 bit, τ_*psc,biol*_ = 65.5 s,τ_*psc,tech*_ = 437 μ*s*).

**Table 7:** **High sample rate, low resolution comparison**.

	**This work**	**Weaver et al., 2011**	**Jain et al., 2012**
Technology	180 nm	90 nm	130 nm
VDD	1.8 V	0.7 V	1.3 V
Power	160 mW	1.11 mW	4.0 mW
Area	2.92 mm^2^	0.18 mm^2^	0.38 mm^2^
*f*_*sample*_	12 MHz	21 MHz	15.625 MHz
ENOB/SNR	7.5 bit	5.8 bit	11.1 bit
FOM	74 pJ/conv-step	0.95 pJ/conv-step	0.11 pJ/conv-step
Architecture	Neuromorphic parallel	Synthesized flash	high-speed DSM

**Table 8:** **Medium sample rate, medium resolution comparison**.

	**This work**	**Han et al., 2013**	**Perez et al., 2011**
Technology	180 nm	180 nm	180 nm
VDD	1.8 V	0.45 V	1.5 V
Power	160 mW	1.35 μW	0.14 mW
Area	2.92 mm^2^	–	0.48 mm^2^
*f*_*sample*_	750 kHz	200 kHz	200 kHz
ENOB/SFDR	11.5 bit	8.3 bit	13.6 bit
FOM	74 pJ/conv-step	0.022 pJ/conv-step	0.056 pJ/conv-step
Architecture	Neuromorphic parallel	SAR	CT-DSM

**Table 9:** **Low sample rate, high resolution comparison**.

	**This work**	**Chae et al., 2013**	**Liu et al., 2013**
Technology	180 nm	160 nm	mixed 500 and 180 nm
VDD	1.8 V	1.8 V	3.3 V
Power	160 mW	6.3 μW	0.28 mW
Area	2.92 mm^2^	0.38 mm^2^	1.14 mm^2^
*f*_*sample*_	730 Hz	25 Hz	10 kHz
ENOB/SFDR	21.5 bit	19.8 bit	17.4 bit
FOM	74 pJ/conv-step	0.28 pJ/conv-step	0.16 pJ/conv-step
Architecture	Neuromorphic parallel	Zoom (SAR+DSM)	Incremental DSM

A common figure of merit (FOM) is used in the comparison that normalizes resolution, sampling rate and power (Walden, 1994). The state-of-the-art is chosen from the continuously updated survey in Murmann (2013). There is some debate whether power and area of the digital blocks of an ADC should be counted, as DSM comparisons usually leave out the decimation filter and other ADCs do not count their anti-aliasing filter (Murmann, 2013). However, our opinion is that since the digital components are an integral part of regular DSM ADCs and also of the presented NEF ADC, they should be included for a fair comparison, i.e., our comparison is based on 160 mW. This should be taken into account when viewing the FOM comparison with Jain et al. (2012) in Table 7 and with Perez et al. (2011) in Table 8.

## 4. Discussion

### 4.1. NEF in a general neuromorphic VLSI context

NEF has recently attracted significant interest from the neuromorphic community, with e.g., an implementation on Neurogrid (Choudhary et al., 2012). It exhibits several features of interest to engineers. Using it, one can engineer a neural system with a target reliable behavior based on unreliable elements. The target behavior can range from building blocks familiar to an engineer, such as control systems or filters (Dethier et al., 2013), up to abstract cognitive functions (Eliasmith, 2007). This paper has highlighted another useful aspect: NEF makes it easy to cross timing domains from asynchronous to synchronous and from analog to digital value representation. Traditionally, this has been one of the major bottlenecks when interfacing neuromorphic systems to more conventional processing units.

The other main challenge of neuromorphic engineering, i.e., achieving biological real time operation (Giulioni et al., 2012), could also be alleviated by NEF. By not representing the system variables directly as spikes, but rather abstracting the single pulses to a time-varying system state vector or scalar variable (Equation 5), the underlying neurons can be dictated by CMOS constraints (i.e., can be operated faster), while the state vector changes could be slower, i.e., able to interact with the outside world in biological real time. By adding this layer of abstraction on top of the neuromorphic network, the CMOS speed advantage can be utilized for e.g., a higher fidelty computation and/or representation of the system state variables, as shown in this paper. This layer of abstraction can also be used to transmit computational variables between neuromorphic units in a more CMOS-friendly fashion. Traditionally, states of neural networks are communicated by the single underlying spikes, requiring large bandwidths in FPGA-based spike routers (Hartmann et al., 2010) or even dedicated IC solutions (Scholze et al., 2011). By abstracting the single pulses to a time-varying digital state, bandwidth can be reduced significantly.

### 4.2. Other neuromorphic ADCs

There are a number of groups that have built ADCs based on neural networks. Table 10 gives an overview of the salient features of these ADCs.

**Table 10:** **Comparison of various neuromorphic ADC concepts**.

**References**	**Description**	**Most similar conventional architecture**	**Parallel/ serial**	**Required analog precision / required design effort**	**Config**.	**Power**	**Sensor fusion possible**
This work	digital decoding of analog input from neuron population signals	Flash and feedforward oversampling	Parallel	Very low / low, repetitive neuron circuit	Rate and resolution	x10 more power than best reported conventional	Yes
Tapson and van Schaik, 2012	Parallel noise shaping network with lateral inhibition	DSM	Parallel	Low / low, repetitive neuron circuit	rate and resolution	No data	No data
Chande and Poonacha, 1995	Binary threshold neurons in a weighted MSB to LSB decoder network	Successive approximation	Serial	Equal to ADC resolution / low, repetitive neuron circuit	resolution	No data, but likely comparable to median conventional	Yes
Yang and Sarpeshkar, 2006	Time-domain pipeline architecture, with neurons handling time domain processing	Pipeline	Serial	Equal to ADC resolution / high, numerous handcrafted components	No	on par with best reported conventional, subthreshold operation	No

Some of those use time-invariant threshold neurons in architectures derived from conventional flash or pipeline ADCs (Chande and Poonacha, 1995). Neuromorphic principles have also been used to convert conventional architectures into the time domain. For example, Yang and Sarpeshkar (2006) show a pipeline ADC composed of Integrate-and-Fire (IAF) neurons that transfers the AD conversion into the time domain. While the use of subthreshold operation in Yang and Sarpeshkar (2006) makes for a very power efficient pipeline design, the entire design is targeted at a single application, without the wide configuration ability of the NEF ADC. For example, a higher resolution can only be achieved in the design of Yang and Sarpeshkar (2006) by increasing the complexity and power draw of the comparator. Also, a higher sample rate is only achievable through a non-subthreshold-operation of the neurons, loosing the energy advantage.

In both Chande and Poonacha (1995) and Yang and Sarpeshkar (2006) the performance of the design is ultimately limited by the precision of its handcrafted building blocks. Thus, no significant advantage is gained compared to conventional ADCs. In particular, both the above ADCs do not use the high parallelism of neural networks to increase robustness and/or conversion speed or precision. In contrast, another family of devices uses the noise shaping effect that a group of neurons achieves when recurrently inhibitory connected (Watson et al., 2004; Tapson and van Schaik, 2012). Here, the signal is represented robustly across a neuron population, i.e., the overall network activity is modulated by the signal (Mayr et al., 2009). The distribution across a neuron population even allows representation of signals above the intrinsic frequency of single neurons (Spiridon and Gerstner, 1999). One main drawback is that some of these architectures are unstable. There is also no fully established method to extract the digital output signal from such a network (Mayr and Schüffny, 2005).

### 4.3. NEF as an analog-digital converter

The NEF ADC shares some characteristics with different conventional ADCs. For instance, time-domain ADCs also integrate the input signal to arrive at analog to time conversion that can then be digitized (Yang and Sarpeshkar, 2006). ADCs that oversample the input signal, such as the DSM mentioned in the introduction, also digitize an input signal with high frequency and low initial resolution. Similar to the NEF ADC, they build up resolution by removing high-frequency components with a filter. Also similar to a DSM, for most applications the NEF ADC does not require an analog Nyquist filter due to the low pass filter characteristic of the neurons and the PSC filter. The NEF ADC also shares some characteristics with flash ADCs, as both use a large parallelism of elements to arrive at a coarse fast quantization. Similar to the NEF ADC, some flash ADCs also rely on statistical deviation of elements for their quatization curve Weaver et al., 2011).

The comparison across Tables 7–9 shows that in terms of absolute figures of sample rate and bit resolution achieved, the NEF ADC is competitive. However, it underperforms quite severly with regard to area and power, see the FOM comparison.

The major part of the area of the NEF ADC is spent on the digital building blocks, letting it benefit significantly from technology scaling. Conventional ADCs do not shrink well due to their usually significantly larger portion of analog circuitry. Thus, the area comparison would look decidedly different in e.g., a 28 nm technology, where the digital blocks would only occupy approx. 0.080 mm^2^. Also, a large fraction of the digital area is spent on the conservative choice of the decoder weight resolution, the large width of the decaying accumulator and the reconfiguration options. Thus, a more dedicated, less configurable design would realize additional area savings. The analog neurons can also be shrunk with the technology node, as this increases their speed and amplifies their mismatch, both desirable properties for the NEF ADC.

Pushing the power consumption of the NEF ADC into a competitive range is harder than for the area. However, as the design of the NEF ADC is intended as a proof-of-principle, no effort has been spent on power optimization. Especially the neuron power draw is quite excessive, with its multiple current paths from VDD to ground. More that 80% of its power draw is not spent on charging the membrane or for switching, but in the offset and gain error stages. Due to downscaling, future neurons in smaller technologies may offer the same variation with significantly less involved circuits, i.e., less power budget. The digital circuitry has also not been optimized for low power draw. Since the NEF is robust to small timing variations in its pulses, the initial digital building blocks such as the decoder weight readout and adder tree could be run asynchronously, only synchronizing directly before the decay register. This would save significant power in the clock tree. For overall clocking, energy-efficient variable clock generators (Eisenreich et al., 2009) could be used to adjust the operating frequency of the system, making a system possible that offers the same resolution at different sample rates, similar to (Yip and Chandrakasan, 2011). Also, the multiple configuration options and corresponding bit widths at all stages add to the power draw. Here, gating techniques that shut off parts of the circuitry not needed for a given configuration have to be explored.

In terms of absolute performance figures, Table 9 shows that the NEF ADC may be especially competitive when it comes to achieving very high resolution digitization, as resolution can be achieved cheaply by digitally averaging over a longer time span. This aspect will be preferentially evaluated once the hardware is available.

However, while a one-to-one comparison of the NEF ADC with conventional ADC is informative, it was not the single design target. The main advantages of the NEF used as an ADC are the following:

- In the NEF ADC, the signal is represented in a robust way across a neuron population (see Table 6). Since the network is purely feed-forward, stability is not an issue.
- NEF makes little demand on the specific transfer characteristics of the analog neurons, and the encoder network uses binary weights. Accordingly, no high-fidelity, complex analog circuits are required anywhere in the system. The handcrafted analog circuits usually needed for an ADC are reduced to two simple neuron circuits, that are multiply instantiated.
- A large part of the processing is carried out in digital, making technology scaling very attractive and enabling design transfer across technologies with minimum effort.
- The possibility of adjusting the transfer characteristic, resolution and sample rate at runtime make for a very flexible system. In addition, the NEF framework incorporates a simple method to input several signals into this network and do computation with them for e.g., sensor fusion.

In addition, NEF represents a theoretically well-explored paradigm, coming complete with a mathematically rigorous method for high-fidelity extraction of the original signal (Eliasmith and Anderson, 2004). Scaling and signal representation behavior necessary to achieve a given target ADC characteristic has been partially established in Choudhary et al. (2012) and treated in depth in this manuscript.

### 4.4. Limits of the NEF ADC resolution

The INL plots of section 3.2 illustrate how insufficient decoder weight resolution, insufficient neuron number or tuning curve variation (represented by setting decoder weights zero) or insufficient tuning curve characterization (represented by perturbed decoder weights) can negatively influence the static INL. Especially for the case of perturbed decoder weights, the ENOB does not provide sufficient characterization of the ADC characteristic, as it stays virtually constant. The INL plots on the other hand provide a clear indication that static INL dominates dynamic INL (i.e., the INL caused by incomplete filtering as seen in the waveform-based INL in Figure 8). As can be seen from Table 2, increasing the number of neurons increases resolution only sublinear, while power draw increases linearily. Thus, an ideal NEF ADC should be operated at the border between the dynamic INL and the static INL (also Figure 8). In other words, tuning curve variation, decoder weight resolution and especially neuron number should just be sufficient for the target INL, with τ_*PSC*_ chosen such that the remaining pulse noise is on the same order as the static INL.

The above is valid if the NEF ADC is built for a single conversion characteristic. In contrast, when using the NEF ADC over a wide range of possible τ_*PSC*_, there are two different options. Either the number of neurons is chosen very large so that even for the high resolution at large τ_*PSC*_, a sufficiently linear overall transfer characteristic can be constructed from the neuron tuning curves. However, this implies that at small τ_*PSC*_, the number of neurons is far in excess of those needed and the NEF ADC is dominated by pulse noise. The second option would be to choose the number of neurons only sufficient for linearity at small τ_*PSC*_, i.e., at low resolutions. At high resolutions (large τ_*PSC*_), the static INL would intentionally dominate. To still achieve linearity, the digital output codes of the low pass filter would be passed through a look-up-table containing the inverse of the static INL curve.

### 4.5. Outlook

In the current version, the NEF ADC still has a number of drawbacks. It is very susceptible to temperature and VDD variation. The transfer characteristic must thus ideally be measured for all these operating conditions and stored, or a constant on-line characterization has to be carried out. Built-in self-tests (BIST) such as Flores et al. (2004) look promising, as they would allow enhancing the NEF ADC with a constant self-monitoring at very little reduction of usable sample rate. Especially digital-heavy versions of BIST could be incorporated with little detriment in design time, as most of the functionality would be synthesizable. The area overhead would also be minimal if the NEF ADC is used as part of a larger digital system where existing compute resources could be reused for BIST (Flores et al., 2004).

A second, more experimental approach might be to adjust the decoder weights online via neuromorphic means, such as synaptic plasticity. NEF has been shown to be amenable to supervised biologically plausible plasticity rules which have as supervisory input the overall transfer characteristic (Bekolay et al., 2013). This plasticity could act either in the analog domain as adjustable factor in the single neuron processing chains, or it could act directly on the digital decoder weights. A candidate plasticity rule that can be configured for a wide range of behavior, i.e., for different compensation or decoder characteristics, has recently been demonstrated (Mayr and Partzsch, 2010) and implemented efficiently in analog CMOS hardware (Mayr et al., 2010a). Digital plasticity rules have been shown e.g., on the Spinnaker system (Jin et al., 2010).

The main point for future work, however, will be to take advantage of the computational capability inherent in NEF. In this paper, NEF has been reduced to a linear representation of a single variable. We will explore various non-linear ADC characteristics and joint conversion of multiple inputs, offering complex sensor fusion and feature extraction (König et al., 2002; Mayr and Schüffny, 2007). Beyond the usage as ADC, the NEF could pave the way toward a future mixed-signal, mixed neuromorphic/conventional system on chip. The NEF could take various elements (regular CMOS, memristors (Jo et al., 2010; Ou et al., 2013), other nanoscale elements) and engineer a system with a set of target computations based on these elements. As demonstrated, such a framework can easily cross the barrier between asynchronous and synchronous systems as well as between analog and digital domains, doing the signal reconstruction either digitally as demonstrated here or via compact, configurable analog PSC circuits (Noack et al., 2010). Signal reconstruction could be via a decoder learned in memristors (Mayr et al., 2012). Thus, one could employ each type of system/device where it is most beneficial and arrive at an amalgan of the state of the art in the neuromorphic discipline, the digital/analog CMOS discipline and in nanodevice systems.

### Conflict of interest statement

The authors declare that the research was conducted in the absence of any commercial or financial relationships that could be construed as a potential conflict of interest.
